# Corrosion Behavior of Nacre-Inspired (TiBw-TiB_2_)/Al Composites Fabricated by Freeze Casting

**DOI:** 10.3390/ma17112534

**Published:** 2024-05-24

**Authors:** Jidong Zhang, Mingfang Qian, Ruiqing Yang, Feng Yu, Xuexi Zhang, Zhenggang Jia, Aibin Li, Guisong Wang, Lin Geng

**Affiliations:** 1 School of Materials Science and Engineering, Harbin Institute of Technology, Harbin 150001, China; 21b909040@stu.hit.edu.cn (J.Z.); zgjia@hit.edu.cn (Z.J.); aibinli@hit.edu.cn (A.L.); wangguisong@hit.edu.cn (G.W.); genglin@hit.edu.cn (L.G.); 2 The National Key Laboratory for Precision Hot Forming of Metals, Harbin Institute of Technology, Harbin 150001, China; 3 Northeast Light Alloy Co., Ltd., Harbin 150060, China; yangruiqing@nela.com.cn (R.Y.); tczonghe@nela.com.cn (F.Y.); 4 State Key Lab of Advanced Welding and Joining, Harbin Institute of Technology, Harbin 150001, China

**Keywords:** freeze casting, nacre-like architecture, (TiBw-TiB_2_)/Al composites, corrosion behavior

## Abstract

Nacre-inspired metal matrix composites have received much attention due to their excellent deformation coordination ability, which can achieve the synergy of strength and ductility. The preparation of nacre-like Al matrix composites by freeze casting has been a promising application, but the continuous ceramic-rich layer affects the corrosion resistance of the composites, facing complex corrosion problems during service. In this work, the microstructure and corrosion behavior of the nacre-inspired (TiBw-TiB_2_)/Al composites fabricated by freeze casting and squeeze casting were systematically studied. The results indicated that the Al layers and ceramic-rich layers had little change, about 35 μm and 31 μm, respectively, with an increasing ratio of the Ti/TiB_2_. Meanwhile, a high Ti/TiB_2_ ratio resulted in an increase in the Fe-Ti intermetallic phases, which was detrimental to the corrosion performance of the composites and was prone to pitting. The electrochemical test results showed that the 3Ti7TiB_2_ composite had the lowest corrosion current density (15.9 μA) and intergranular corrosion depth (231 μm), indicating that it had the best corrosion resistance, which can be attributable to its stable and dense passivation film. Two different corrosion phenomena during the intergranular corrosion test existed in the present nacre-inspired (TiBw-TiB_2_)/Al composites: intergranular corrosion in the Al matrix layer and pitting corrosion in the ceramic-rich layer. Among all the composites, the corrosion depth of the 3Ti7TiB_2_ composite was the smallest and significantly less than that of the 2024Al alloy. In addition, the continuous ceramic-rich layer acted as a corrosion channel during corrosion, significantly degrading the corrosion resistance of the nacre-like Al composites.

## 1. Introduction

Due to the light weight, good ductility, and excellent corrosion resistance, Al and its alloys have attracted much attention, and thus are widely used in various fields, such as armor, automotive, and transportation [1,2,3]. Unfortunately, the limited strength and wear resistance restrict their applications. To overcome this dilemma, numerous Al matrix composites (AMCs) reinforced with various ceramic particles such as Al_2_O_3_, SiC, TiB_2_, B_4_C, and TiC [4,5,6] have been developed due to their high wear resistance, high specific modulus, and low thermal expansion [7,8]. Over the past decades, researchers have been trying to improve the homogeneous distribution of reinforcements in AMCs to create high-performance particle-reinforced AMCs. However, the homogeneous distribution state involved the difficulty to address the trade-off between strength and ductility [9,10,11]. Fortunately, biomaterials with synergistic strength and ductility provide us with a wealth of inspiration for the preparation of high-performance AMCs.

Recently, the AMCs with bionic architecture, such as nacre-like [12], tubular and lamellar hybrid [13], gradient [14], and bimodal [15,16] structures with superior strength as well as ductility, have been developed. For example, Li et al. [12] prepared Graphene/Al composites with a nacre-like structure, which exhibited a 50% increase in strength with almost no decrease in ductility (fracture elongation only decreased from 5.37% to 5.30%). Yang et al. [13] developed Al_2_O_3_/Al composites with tubular and lamellar hybrid structures, exhibiting superior mechanical properties, such as fracture toughness and flexural strength. Ma et al. [15] fabricated carbon nanotubes (CNTs) and reinforced CNTs/2009Al with a bimodal structure by vacuum hot press sintering, which showed a simultaneous increase in tensile strength and ductility of 2% and 88%, respectively, compared to the uniform CNTs/2024Al composite counterpart. The improved mechanical properties can be ascribed to the suppressed strain localization, and the formation of geometrically necessary dislocations between coarse grain zones and fine grain zones. Up until now, the studies on the Al composites with heterogeneous architecture have focused on the preparation process, microstructure, and mechanical properties, while the service environment in which the Al composites are used is becoming increasingly complex, requiring composites to have not only superior mechanical properties but excellent corrosion resistance [17,18]. It is therefore of great interest to investigate the service performance of Al composites, which is expected to enable their application in corrosive environments. There are many factors that affect the corrosion resistance of the Al composites, including the type, distribution, and volume fraction of the reinforcements [19,20,21]. Chen et al. [19] found that the intergranular corrosion resistance of the TiB_2_/7075Al composites improved significantly with the addition of the TiB_2_ particles, but the electrochemical corrosion properties of the composites were first weakened and then improved with the increase in the TiB_2_ volume fraction. Keshavarz et al. [22] prepared ZrO_2_/Al, graphite/Al, and (ZrO_2_ + graphite)/Al composites by friction stir processing. They found that the corrosion resistance of the ZrO_2_/Al and graphite/Al composites was better than that of the Al alloy. In addition, the graphite/Al composites have the lowest corrosion rate and pitting susceptibility compared to the ZrO_2_/Al and (ZrO_2_ + graphite)/Al composites. Zhao et al. [20] investigated the effect of the TiC particle distribution on the corrosion resistance of the TiCp/7050 composites, and found that the corrosion resistance of composites with homogeneously dispersed TiC is superior to that of composites with network or chain-like particles.

Titanium diboride (TiB_2_) ceramics have remarkable strengthening effects in Al matrix composites due to their high modulus (550 GPa), ultra-high hardness (35 GPa), and excellent thermal stability [23,24]. Therefore, numerous efforts have been made to fabricate high-performance TiB_2_/Al composites. A typical example is a bimodal TiB_2_/Al composite with excellent properties, prepared by powder metallurgy and hot extrusion, which achieves precise control of the volume fraction of ultrafine and coarse grains [25]. However, little information has been provided on the corrosion resistance of TiB_2_/Al composites, especially bio-inspired TiB_2_/Al composites. Recently, freeze casting techniques, including preform preparation and melt infiltration, have been widely used for the preparation of nacre-like Al composites. However, the temperature used to prepare TiB_2_ preforms is often high, exceeding 1700 °C. The in situ reaction (Ti + TiB_2_ → 2TiBw) can significantly reduce the sintering temperature of the preforms, which is around 1200 °C. In addition, the TiBw introduced by the in situ reaction has a coefficient of thermal expansion similar to that of the Al matrix in addition to its high hardness and stiffness, and does not react with the Al matrix at high temperatures. In this study, a novel nacre-like (TiBw-TiB_2_)/Al composite was fabricated by freeze casting and squeeze casting. The effect of the Ti/TiB_2_ ratio on the microstructure and corrosion resistance of the nacre-like (TiBw-TiB_2_)/Al composites was investigated systematically. Electrochemical and intergranular corrosion tests have been used to evaluate the corrosion properties of the composites, and the corresponding corrosion mechanisms have been elucidated.

## 2. Materials and Methods

### 2.1. Preparation of Bio-Inspired (TiB_2_-TiBw)/2024Al Composites

**Figure 1:**
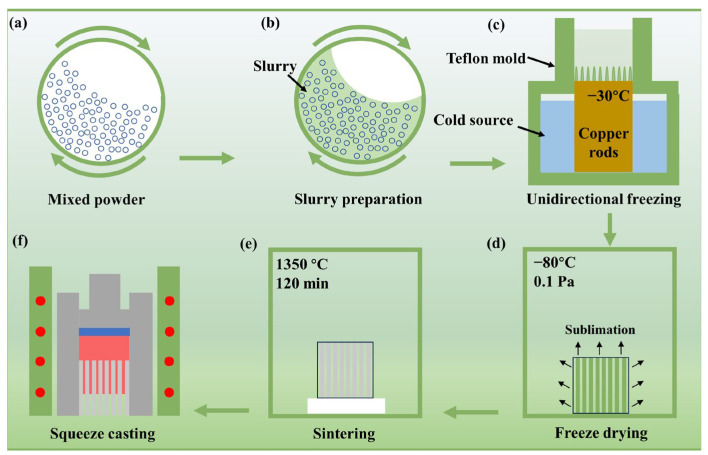
A schematic diagram for the preparation of the nacre-like (TiBw-TiB_2_)/Al composites.

The raw materials used to fabricate the (TiB_2_-TiBw) preforms are commercial TiB_2_ powder (D_50_ = 5 μm, purity ~ 99.8%) and Ti powder (D_50_ = 10 μm, purity ~ 99.9%) provided by Shanghai Buwei Applied Materials Technology Co., Ltd., Shanghai, China. The TiB_2_ and Ti powders have chemical compositions of TiB_2_-0.02Fe-0.01Ca-0.05Mg-0.01Cu-0.02Mn-0.01Na-0.01Zn (wt.%) and Ti-0.03Fe-0.01Mg-0.01Mn-0.02Si-0.02C-0.01N (wt.%), respectively, based on the powder specification from the powder supplier. To prepare the slurries, the TiB_2_ and Ti powders were firstly mixed by ball milling to obtain a homogeneous mixture (Figure 1a), and then the 20 vol.% mixed powders, 1 wt.% PVA, and 0.8 wt.% Xanthan gum were added to deionized water and stirred well (Figure 1b). The homogeneous slurries were subsequently poured into a cylindrical Teflon mold placed on a cooling source at −30 °C until complete solidification (Figure 1c). To sublimate the ice, the frozen samples were placed in a freeze dryer maintained at −80 °C, 0.1 Pa, for 72 h (Figure 1d). After freeze–drying, the green preforms were sintered at 1350 °C for 2 h, followed by furnace cooling (Figure 1e). 

The bio-inspired (TiB_2_-TiBw)/2024Al composites were fabricated by squeeze casting, where the molten 2024Al was squeezed into the porous TiB_2_-TiBw preforms. The preforms were placed in a cylindrical heat-resistant steel mold, and then heated to 580 °C using a ring heating furnace. The 2024Al ingots were placed in a ceramic crucible, heated to 750 °C, held for 60 min, and then poured into the heat-resistant steel mold. Subsequently, a pressure of 50 MPa was applied until the molten Al was solidified (Figure 1f). To obtain high-performance composites, the bio-inspired (TiB_2_-TiBw)/2024Al composites were solution treated at 500 °C for 2 h, then quenched in water and artificially aged at 160 °C for 16 h. 

### 2.2. Corrosion Tests 

A potentiodynamic polarization test was employed to evaluate the corrosion behavior. For electrochemical tests, a three-electrode cell system was used, where Al composites (1 × 1 × 0.3 cm^3^) were regarded as working electrodes; a platinum flake with a size of 1 × 1 × 0.1 cm^3^ was used as the counter electrode, and a saturated calomel electrode was utilized as the reference electrode. The electrochemical tests were carried out in a 3.5 wt.% NaCl solution at ambient temperature on an electrochemical workstation (Bio-Logic, SP-300, Claix, France). To obtain stable open circuit potential (OCP), all the samples were immersed in a 3.5 wt.% NaCl solution for 60 min prior to electrochemical tests, and then the OCP was measured for 1800 s. Subsequently, the electrochemical impedance spectroscopy (EIS) was performed with a scan frequency range of 10^5^ to 10^−2^ Hz and an AC amplitude of 10 mV under the OCP conditions. In addition, the potentiodynamic polarization tests were carried out vs. SCE between −1.5 and −0.5 V, with a scan rate of 0.1 mV/s.

The intergranular corrosion test was carried out based on the GB/T 7998-2005 standard [26] in a mixture solution of 57 mg/mL of NaCl and 10 mL/L of hydrogen peroxide (H_2_O_2_) at 35 °C for 12 h. For the intergranular corrosion test, the composite samples were placed in a 30 wt.% HNO_3_ solution, and then put into the corrosive solution. To evaluate the resistance to intergranular corrosion, the composites were cut along the longitudinal section and the penetration depths of the intergranular corrosion were compared.

### 2.3. Characterization Analysis

An X-ray diffraction (XRD) test was performed to examine the phases in the TiB_2_-TiBw preforms and composites. The microstructure and corrosion surface morphologies of the composite were analyzed using scanning electron microscopy (SEM, Hitachi, S-4800, Tokyo, Japan) equipped with an energy-dispersive spectrometer (EDS). The thickness of the Al and ceramic-rich layers was measured using Nano Measurer software (version 1.2), and more than 200 layers were measured to ensure the accuracy of the data. The composition on the corrosion surface was also analyzed via X-ray photoemission spectroscopy (XPS).

## 3. Results

### 3.1. Microstructure Characterization

**Figure 2:**
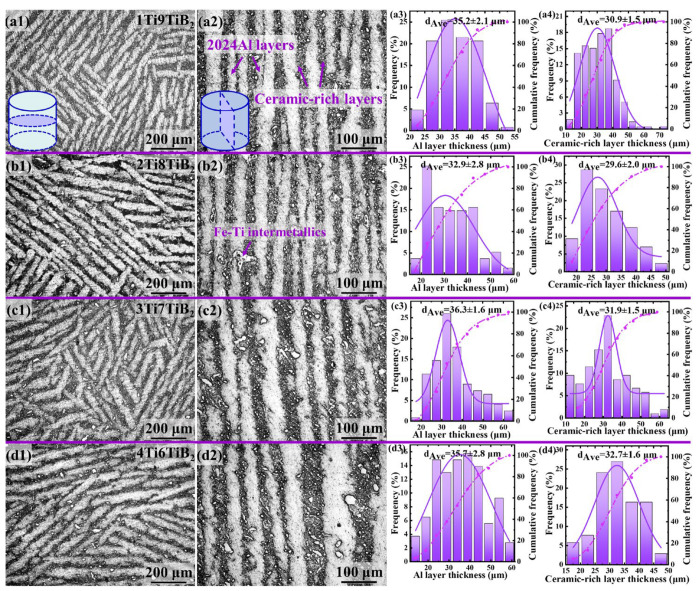
Microstructures of the nacre-like (TiBw-TiB_2_)/2024Al composites with various compositions of 1Ti9TiB_2_, 2Ti8TiB_2_, 3Ti7TiB_2_, and 4Ti6TiB_2_. (**a1**–**d1**) and (**a2**–**d2**) are optical images showing morphologies of the composites perpendicular and parallel to ice-crystal growth directions, respectively; (**a3**–**d3**) and (**a4**–**d4**) are statistic thicknesses of Al and ceramic-rich layers, respectively.

Figure 2 shows the microstructure of the nacre-like (TiBw-TiB_2_)/2024Al composites with initial Ti contents of 10%, 20%, 30%, and 40% (denoted as 1Ti9TiB_2_, 2Ti8TiB_2_, 3Ti7TiB_2_, and 4Ti6TiB_2_, respectively). In the optical microscope images of the composites Figure 2(a1–d1,a2–d2), the bright layers correspond to the 2024Al matrix, while the dark layers to the ceramic-rich areas. Obviously, few pores are visible in the composites, indicating the complete infiltration of the aluminum matrix in the preforms under the applied high pressure during squeeze casting. The molten Al alloy filled not only the interspace between the ceramic-rich layers, but also the ceramic-rich layers. Thus, an interpenetrating structure was successfully formed via low-temperature sintering of the preform combined with high-temperature pressure infiltration.

The histograms of layer thickness of the Al matrix and ceramic-rich layer are calculated from the length of the sections perpendicular and parallel to the ice growth direction, as shown in Figure 2(a3–d3,a4–d4). The average thickness of Al layers (*t_m_*) is 35.2, 32.9, 36.3, and 35.7 μm in 1Ti9TiB_2_, 2Ti8TiB_2_, 3Ti7TiB_2_, and 4Ti6TiB_2_ composites, respectively. Correspondingly, the average thickness of ceramic-rich layers (*t_c_*) is 30.9, 29.6, 31.9, and 32.7 μm, respectively. It is noted that the four kinds of composites showed a similar thickness ratio (*t_m_/t_c_*) ~ 1.1. 

**Figure 3:**
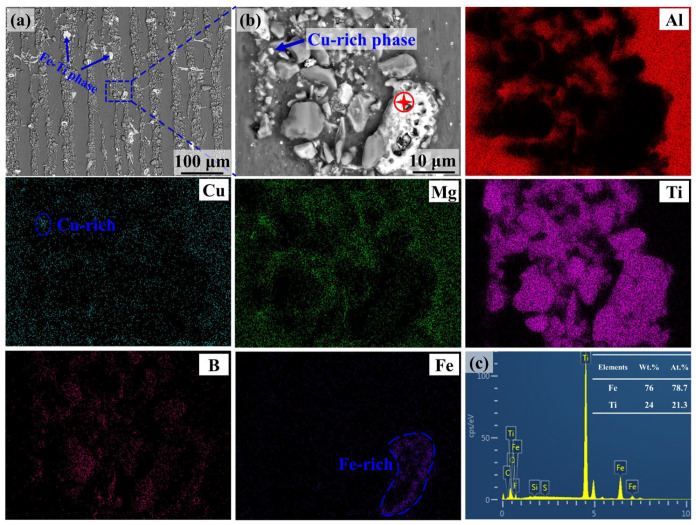
Back scanning electronic (BSE) image and energy-dispersive spectrum (EDS) showing distribution of Fe-Ti phase in 1Ti9TiB_2_ composite. (**a**) Fe-Ti intermetallic phase distributes in ceramic-rich layers; (**b**) enlarged image of Fe-Ti phase and corresponding element mapping. (**c**) EDS result of phase marked red in (**b**).

In addition, the content of the reaction products in the nacre-like composites increases with an increasing ratio of Ti and TiB_2_, as shown in Figure 2(a2–d2). A typical micrograph showing the reaction products is shown in Figure 3a. By a BSE and EDS point analysis, the reaction products are determined to be an Fe-Ti intermetallic phase (Figure 3b,c), which is considered to be formed by the reaction of the residual titanium in the preforms with a small amount of Fe in the molten 2024Al alloy during the pressure infiltration process. Such a brittle phase is often detrimental to the mechanical properties of the composites. According to the EDS mapping results, a few Cu-rich phases remaining in the ceramic-rich layers can be found due to the limited diffusion capacity of the Cu element in the reinforcement-rich areas [18,21]. 

**Figure 4:**
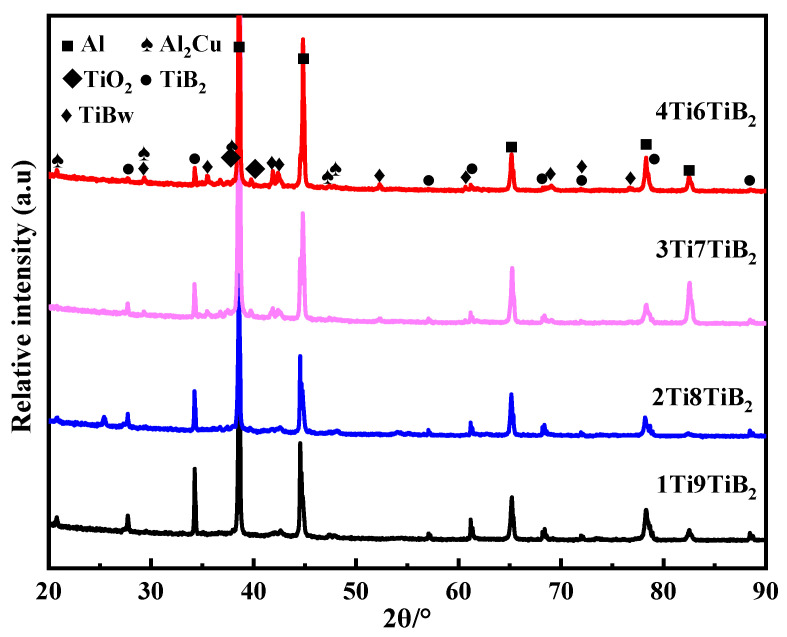
XRD results of the nacre-like (TiBw-TiB_2_)/2024Al composites with various compositions of 1Ti9TiB_2_, 2Ti8TiB_2_, 3Ti7TiB_2_, and 4Ti6TiB_2_.

To determine the phase composition of the nacre-like (TiBw-TiB_2_)/2024Al composites with different Ti/TiB_2_ ratios, XRD analyses were carried out, as shown in Figure 4. In Figure 4, diffraction peaks of TiBw and Al_2_Cu are detected in all composites, and the intensity of the diffraction peaks becomes stronger with an increasing Ti/TiB_2_ ratio. In addition, the TiO_2_ diffraction peaks appear, which become stronger with an increasing Ti/TiB_2_ ratio as well. The results confirm that, with the increase in the Ti/TiB_2_ ratio, the content of residual Ti increases, which reacts with oxygen (Ti + O_2_ → TiO_2_) during pressure infiltration to form TiO_2_. 

### 3.2. Electrochemical Corrosion Tests

#### 3.2.1. Potentiodynamic Polarization

**Figure 5:**
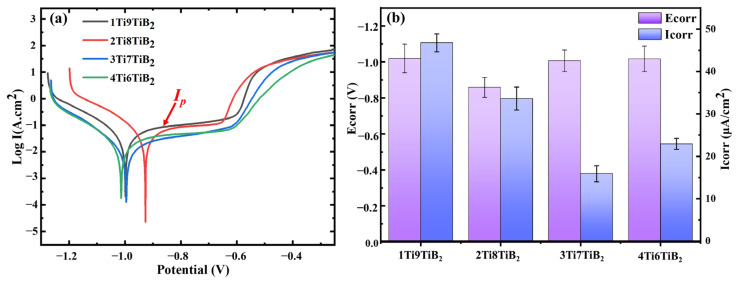
Corrosion character of nacre-like (TiBw-TiB_2_)/2024Al composites with various compositions of 1Ti9TiB_2_, 2Ti8TiB_2_, 3Ti7TiB_2_, and 4Ti6TiB_2_ in 3.5 wt.% NaCl solutions. (**a**) Potentiodynamic cures; (**b**) corrosion potential (*E_corr_*) and current density (*I_corr_*).

The potentiodynamic polarization technique has been extensively used to investigate the corrosion behavior as well as the formation mechanism of the passive/protective film for metals and composites [19,27]. The potentiodynamic polarization results of the nacre-like (TiBw-TiB_2_)/2024Al composites in the 3.5 wt.% NaCl solution are shown in Figure 5a. The corresponding self-corrosion potential (*E_corr_*) and current density (*I_corr_*) are also obtained by the Tafel extrapolation method, as shown in Figure 5b and Table 1**.** Generally, the higher the corrosion potential, the better the corrosion resistance [21,28,29]. In contrast, the lower the *I_corr_*, the better the corrosion resistance of the samples. From Table 1, it is noticed that the *E_corr_* for all composites fluctuates between −0.858 and −1.019 V, in which the 1Ti9TiB_2_ composite has the lowest *E_corr_* (−1.019 ± 0.08 V). This implies that the 1Ti9TiB_2_ composite exhibits the worst corrosion resistance in the 3.5 wt.% NaCl solution. On the other hand, the *I_corr_* of the 1Ti9TiB_2_ composite is highest among all the composites, corresponding to the fastest corrosion rate. Interestingly, the 2Ti8TiB_2_ composite has the largest *E_corr_*, but it does not have the lowest *I_corr_*, while the 3Ti7TiB_2_ composite has the lowest *I_corr_*, which is 66% lower than that of the 1Ti9TiB_2_ composite. Thus, it is difficult to determine which composite has the best corrosion resistance by *E_corr_* and *I_corr_* values.

Passivation current density (*I_p_*), passivation potential (*E_p_*), and the breakdown potential (*E_b_*) are also often used to determine the corrosion resistance of a sample. Here, *I_p_* can be obtained from the horizontal segment shown in Figure 5a. The higher *I_p_* indicates a faster dissolution of the passivation film [25,27,28]. *E_p_* indicates the onset of passivation, where the passivation film begins to form. Thus, a low passivation voltage is favorable for the formation of a passivation film on the sample surface. For the *E_b_*, it indicates the critical potential value for the broken area of the passivation film; the higher it is, the more stable the passivation film formed on the sample surface. Obviously, the 3Ti7TiB_2_ composite has lower *I_p_* and *E_p_* compared to the 2Ti8TiB_2_ composite, indicating that passivation films form more readily on the sample surface and dissolve more slowly. In addition, the *E_b_* of the 3Ti7TiB_2_ composite is higher than that of the 2Ti8TiB_2_ composite. According to the above discussion, we can conclude that the 3Ti7TiB_2_ composite has the best corrosion resistance.

**Table 1:** Electrochemical parameters of nacre-like (TiBw-TiB_2_)/2024Al composites.

Samples	*E_corr_* (V)	*I_corr_* (μA/cm^2^)	*E_p_* (V)	*I_p_* (mA/cm^2^)	*E_b_* (V)
1Ti9TiB_2_	−1.019 ± 0.08	46.75 ± 2.1	−0.921 ± 0.05	58.0 ± 2.5	−0.613 ± 0.03
2Ti8TiB_2_	−0.858 ± 0.06	33.6 ± 2.7	−0.815 ± 0.03	37.3 ± 2.0	−0.661 ± 0.05
3Ti7TiB_2_	−1.006 ± 0.06	15.9 ± 1.9	−0.875 ± 0.03	29.4 ± 1.3	−0.620 ± 0.02
4Ti6TiB_2_	−1.017 ± 0.07	22.9 ± 1.3	−0.934 ± 0.01	28.6 ± 1.1	−0.629 ± 0.03

*E_corr_*—Self-corrosion potential; *I_corr_*—Corrosion current density; *E_p_*—Passivation potential; *I_p_*—Passivation current density; *E_b_*—Breakdown potential.

**Figure 6:**
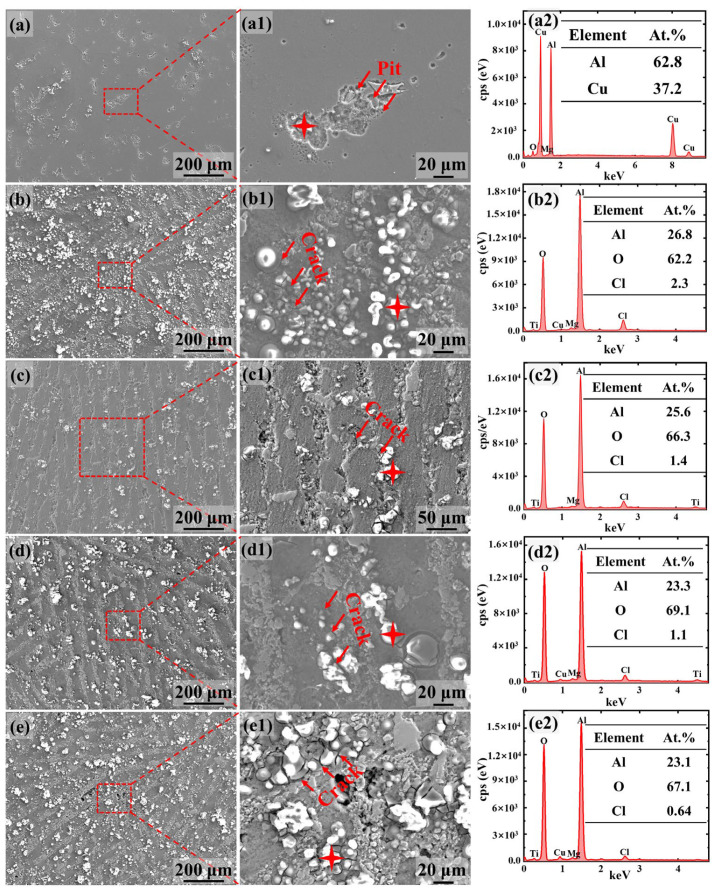
Surface corrosion morphologies of 2024Al alloy and nacre-like (TiBw-TiB_2_)/2024Al composites after potentiodynamic polarization tests. (**a**–**a2**) 2024Al alloy; (**b**–**b2**) 1Ti9TiB_2_; (**c**–**c2**) 2Ti8TiB_2_; (**d**–**d2**) 3Ti7TiB_2_; and (**e**–**e2**) 4Ti6TiB_2_; (**a1**–**e1**) correspond to the red dashed box in (**a**–**e**), and the red arrows indicate the cracks; (**a2**–**e2**) the EDS result of the red four-pointed star in (**a1**–**e1**).

To further investigate the corrosion behavior of the composites during potentiodynamic polarization testing, the corrosion morphologies and products were analyzed by SEM. For the 2024Al matrix alloy, the pitting corrosion character occurred (Figure 6a), developing into corrosion pits around the secondary phase (Al_2_Cu according to EDS results). This indicates that the secondary phase acts as a cathode during corrosion. This is consistent with the previous report that the Al_2_Cu phase is susceptible to corrosion and can act as an initial corrosion site for pitting [30]. 

For the 1Ti9TiB_2_ composite, the pitting occurs almost exclusively in the Al matrix near the ceramic layer, producing many corrosion products (Al_2_O_3_, Al(OH)_3_, and AlCl_3_) (Figure 6(b,b2)). In addition, cracks also form around the corrosion products. Similarly, the pitting corrosion products and the cracks also occur in 2Ti8TiB_2_, 3Ti7TiB_2_, and 4Ti6TiB_2_ composites. However, the content of corrosion products decreases and then gradually increases as the Ti/TiB_2_ ratio increases (Figure 6b–e), in which the 2Ti8TiB_2_ composite shows the smallest content of corrosion products on the surface (Figure 6c). Furthermore, there was severe corrosion in the ceramic-rich layers of the 2Ti8TiB_2_ composites, indicating poor corrosion resistance (Figure 6(c1)). Generally, the formation of corrosion products has a direct effect on the corrosion susceptibility of the composites. A dense, adherent film of corrosion products effectively prevents the penetration of corrosive ions. Conversely, a loose corrosion product film with cracks cannot provide an effective barrier to the diffusion of corrosion ions from the solution into the Al matrix [31]. Furthermore, the formation of cracks is especially detrimental to the corrosion resistance. Obviously, the cracks around the corrosion products formed on the surface of the 3Ti7TiB_2_ composites are relatively few, indicating that the corrosion products on its surface are denser than those of the other composites. According to the potentiodynamic polarization result and the corrosion morphology, we have also shown that the 3Ti7TiB_2_ composite has the best corrosion resistance among all the composites.

**Figure 7:**
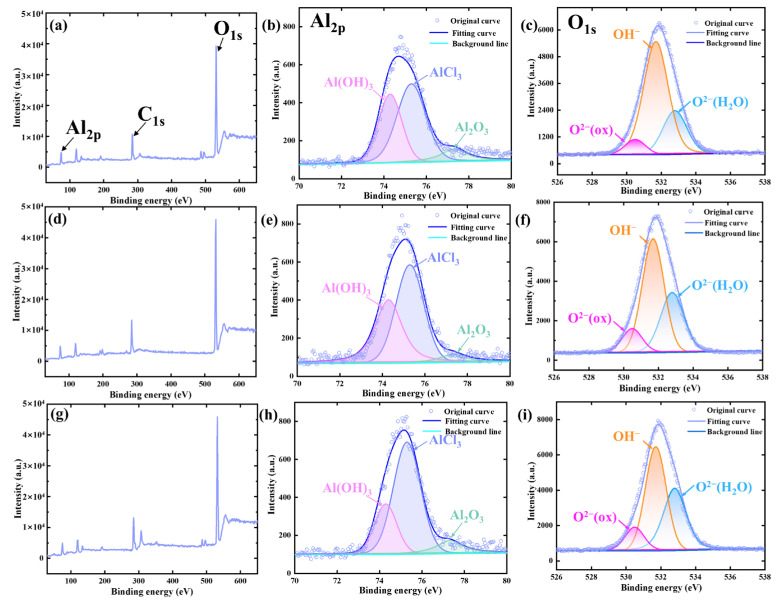
X-ray photoemission spectroscopy (XPS) spectra of nacre-like (TiBw-TiB_2_)/2024Al composites after potentiodynamic polarization testing in 3.5 wt.% NaCl solution. (**a**–**c**) 2024Al, (**d**–**f**) 1Ti9TiB_2_, and (**g**–**i**) 3Ti7TiB_2_.

To reveal the corrosion mechanism of the composites, the corrosion products on the surfaces were characterized using X-ray photoemission spectroscopy (XPS). Figure 7 shows the XPS overview spectra and the high-resolution spectra of the Al_2p_ and O_1s_ on the surface of the 2024Al alloy and 1Ti9TiB_2_ and 3Ti7TiB_2_ composites. The deconvoluted peaks have similar positions, implying that the corrosion products in 2024Al alloy, 1Ti9TiB_2_, and 3Ti7TiB_2_ composite samples are similar. Specifically, the Al spectra can be separated into three peaks, i.e., Al_2_O_3_ (77.38 eV), AlCl_3_ (75.3 eV), and Al(OH)_3_ (74.3 eV), where the Al_2_O_3_ plays an important role in improving the corrosion resistance [29,31]. Similarly, the O spectra can also be separated into three peaks, namely O^2−^ (530.5 eV), OH^−^ (531.7 eV), and H_2_O molecules (532.8 eV), respectively [31]. It has been reported that the O^2−^ tends to form stable phases with the metal elements, whereas OH^−^ is associated with metastable phases [32,33]. According to the results of the Al and O spectra, the corrosion products during corrosion are determined to be mainly Al(OH)_3_ and AlCl_3_. Obviously, the relative peak intensity of the Al_2_O_3_ and Al(OH)_3_ peaks of the 3Ti7TiB_2_ composite is higher than those of the 1Ti9TiB_2_ composite, indicating that a more stable and thicker passivation film has been formed on the surface of the 3Ti7TiB_2_ composite, which is consistent with the results shown in Figure 6(d–d2). 

#### 3.2.2. Electrochemical Impedance Spectroscopy (EIS)

**Figure 8:**
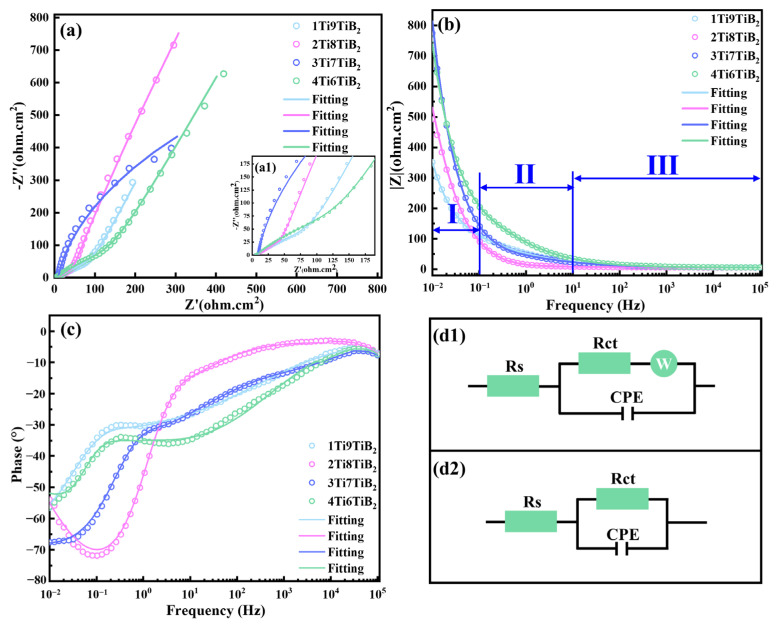
Electrochemical impedance spectroscopy of the nacre-like (TiBw-TiB_2_)/2024Al composites with different Ti/TiB_2_ volume ratios in 3.5 wt.% NaCl solution. (**a**) Nyquist plots; (**a1**) a zoom image of the high-frequency region in (**a**); (**b**,**c**) bode plots; (**d1**,**d2**) equivalent circuit for the fitting.

Although the potentiodynamic polarization test is advantageous for the evaluation of the corrosion properties of samples, the occurrence of cathodic reactions can reduce its accuracy [34,35]. For electrochemical impedance spectroscopy (EIS), it has a low interference signal (only 5–10 mV). Therefore, EIS is used to further investigate the protective properties of the oxide layer formed on the sample surface. Figure 8 shows the EIS results of the nacre-like (TiBw-TiB_2_)/2024Al composites with different Ti/TiB_2_ volume ratios in the 3.5 wt.% NaCl solution. As can be seen in Figure 8a, the 1Ti9TiB_2_, 2Ti8TiB_2_, and 4Ti6TiB_2_ composites have similar Nyquist plots, which can be divided into two stages, including a capacitive loop and an impedance straight line. In contrast, the 3Ti7TiB_2_ only has a capacitive loop, implying that the 3Ti7TiB_2_ composite exhibits the best corrosion resistance since it has the largest radian diameter of the capacitive loop (corresponding to the greatest polarization resistance and therefore the best corrosion resistance). 

The impedance modulus and phase versus frequency curves are shown in Figure 8b,c, respectively. The impedance modulus vs. frequency curves can be divided into three regions (Figure 8b): the low-frequency region (10^−2^–10^−1^ Hz, stage I), intermediate-frequency region (10^−1^–10 Hz, stage II), and high-frequency region (10–10^5^ Hz, stage III). Generally, the impedance modulus value in the low-frequency region corresponds to the polarization resistance of the samples, while the 3Ti7TiB_2_ and 4Ti6TiB_2_ composites have a higher polarization resistance than that of the other two composites (1Ti9TiB_2_ and 2Ti8TiB_2_). For the phase vs. frequency curves (Figure 8c), the maximum phase angles for all samples fall into the range of −50–−70°, in which the 2Ti8TiB_2_ composite shows the largest phase angles. In addition, the phase value of all composites in the high-frequency range approaches zero, and the impedance modulus shows little change with increasing frequency, indicating that only the resistive behavior (i.e., Ohmic potential drop for the electrolytic solution) exists in the frequency region [35,36,37]. 

Based on the impedance behavior described above, the equivalent circuits are fitted for a further interpretation of the electrochemical response of the composites under a given perturbation, as shown in Figure 8(d1,d2). Figure 8(d1) shows an equivalent circuit obtained by fitting the EIS data of the 1Ti9TiB_2_, 2Ti8TiB_2_, and 4Ti6TiB_2_ composites with a constant phase element (CPE), where *R_s_* is the resistance of the electrolyte solution, *R_ct_* is the charge transfer resistance (polarization resistance), and W refers to the electrical double layer and Warburg impedance diffusion, respectively. The equivalent circuit in Figure 8(d2) has been used to fit the EIS data of the 3Ti7TiB_2_ composite. The CPE is defined as *Z_CPE_* = (*jw*)^−*α*^/*Q*, where *Q* represents the capacitance; w and j represent the frequency and current, respectively; and α refers to the charge relaxation coefficient, which is affected by the phase angle and takes values between −1 and 1. Table 2 lists the fitted results according to the EIS. The results show that the polarization resistance of the 4Ti6TiB_2_ composite is higher than that of the 1Ti9TiB_2_ and 2Ti8TiB_2_ composites, which is consistent with the results obtained from the polarization curve test shown in Figure 5 and Table 2.

**Table 2:** Equivalent circuit parameters for impedance spectrum of the nacre-like (TiBw-TiB_2_)/2024Al composites in the 3.5 wt.% NaCl solution.

	1Ti9TiB_2_	2Ti8TiB_2_	3Ti7TiB_2_	4Ti6TiB_2_
Rs (ohm·cm^2^)	6.022 ± 0.005	5.218 ± 0.004	6.95 ± 0.15	5.7 ± 0.4
Rct (ohm·cm^2^)	63.95 ± 0.03	118 ± 3	1914 ± 8	307.6 ± 6
Q (ohm^−1^·cm^−2^·sn)	(5.8 ± 0.1) × 10^−3^	(1.26 ± 0.02) × 10^−3^	(1.65 ± 0.03) × 10^−2^	(4.06 ± 0.05) × 10^−3^
α	0.46 ± 0.02	0.539 ± 0.006	0.819 ± 0.004	0.48 ± 0.03
Ws (S·s^1/2^)	346 ± 6	113.7 ± 3	–	104.9 ± 4

### 3.3. Intergranular Corrosion Testing

**Figure 9:**
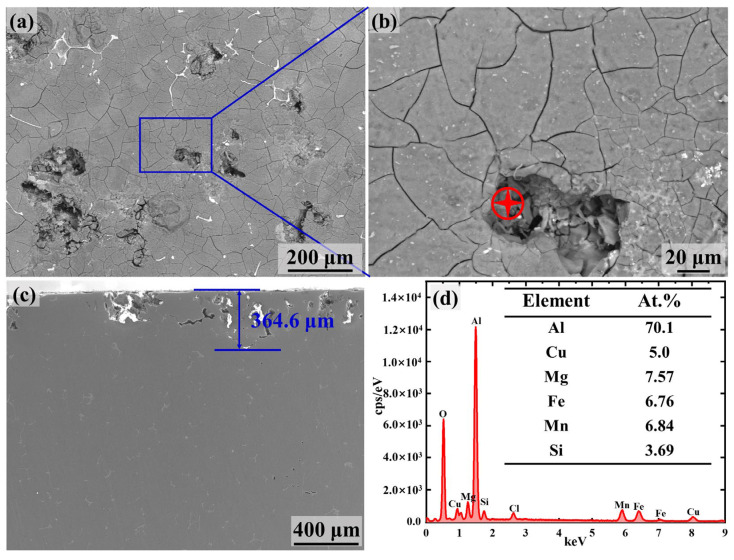
SEM images showing the morphology and composition distribution of the 2024Al alloy subjected to the intergranular corrosion test. (**a**) Morphology of the corroded surface; (**b**) an enlarged image in the solid blue box in (**a**); (**c**) the corrosion depth of the longitudinal section; and (**d**) the EDS results of the corrosion products in the corrosion pits marked red in (**b**).

To evaluate the intergranular corrosion susceptibility of 2024Al and nacre-like (TiBw-TiB_2_)/Al composites, immersion tests were carried out based on the GB/T 7998-2005 standard, after which the corrosion surface and longitudinal section of the corroded samples were characterized to observe the corrosion morphologies and corrosion depth. As can be seen from Figure 9a–c, most of the 2024Al grains are uncorroded, except for intergranular corrosion at grain boundaries and some grains are subjected to pitting corrosion at the grain boundaries. In addition, some second phases are found within corrosion pits (Figure 9b), which are determined by EDS to be the AlCuFeMnSi phase, and their specific compositions are given in the table in Figure 9d. This confirms that the iron-rich particles can act as a cathode and induce corrosion in the surrounding 2024Al matrix alloy. Note that the corrosion products observed in the corrosion pits can be attributed to the complete corrosion of the metal surrounding the intermetallic phases [31]. In order to evaluate the intergranular corrosion susceptibility of the 2024Al, we have characterized the corrosion depth of the longitudinal sections, as shown in Figure 9c, from which it can be seen that the corrosion depth is approximately 364.6 μm.

**Figure 10:**
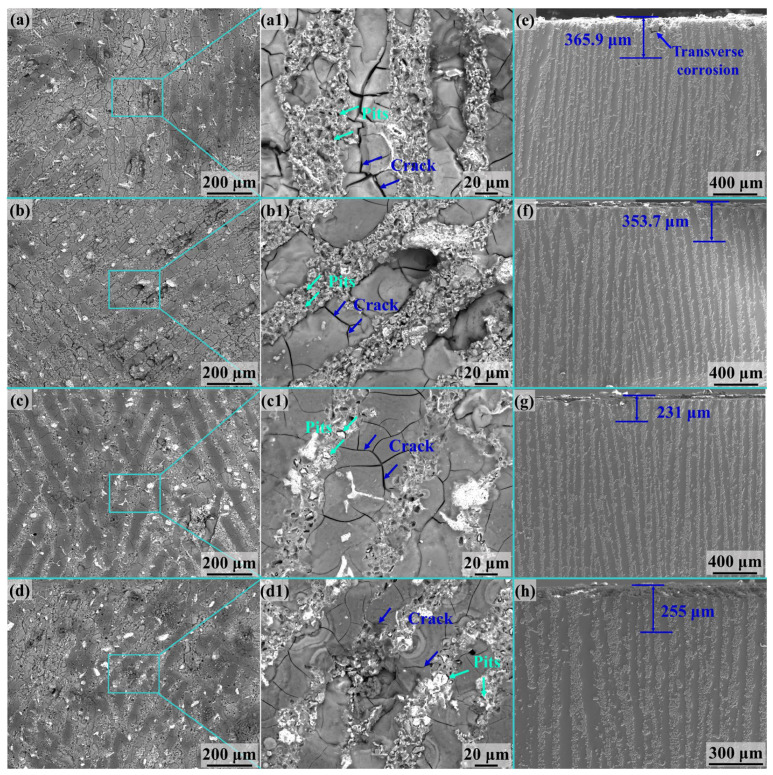
Intergranular corrosion behaviors of the nacre-like (TiBw-TiB_2_)/Al composites. (**a**–**d**) Surface corrosion morphologies and (**e**–**h**) longitudinal sections of the (TiBw-TiB_2_)/Al composites showing the intergranular corrosion depth.

Figure 10 shows the surface corrosion morphology and corrosion depth in the longitudinal section of the (TiBw-TiB_2_)/Al composites during the intergranular corrosion. Unlike 2024Al alloys, intergranular corrosion occurs in the Al layers in the composites and pitting corrosion in the ceramic-rich layers. Figure 10a reveals the intergranular corrosion morphology of the 1Ti9TiB_2_ composites. The corrosion depth of the intergranular corrosion in 1Ti9TiB_2_ composites is comparable to that of 2024Al alloys, and the pitting corrosion occurs in the ceramic-rich layers. From the intergranular and pitting corrosion characteristics of the composites, severe corrosion behavior is associated with ceramic-rich layers. Most of the pitting corrosion appeared in the ceramic-rich layers, indicating that the ceramic is prone to induce pitting (Figure 10(c1,d1)). In addition, the number of corrosion pits within the ceramic-rich layer decreases and the size slightly increases with increasing Ti content (Figure 10(a1–d1)), which is attributed to increasing content of the Fe-Ti phase. 

The intergranular corrosion depths of the nacre-like (TiBw-TiB_2_)/2024Al composites are shown in Figure 10e–h. The intergranular corrosion depth of the 1Ti9TiB_2_ composite is about 365.9 μm, which occurs mainly in the ceramic-rich layers (Figure 10e). In addition, the areas close to the surface of the composite are found to be continuously eroded. From the deeper corrosion areas, it can be seen that transverse corrosion has occurred in the Al matrix layer throughout the Al matrix layer. In comparison, the intergranular corrosion depth of the 2Ti8TiB_2_ composite is 353.7 μm (Figure 10f), which is comparable to that of the 1Ti9TiB_2_ composite. For the 3Ti7TiB_2_ composite (Figure 10g), the corrosion depth is reduced to 231 μm. With a further increase in the addition of Ti particles, the maximum corrosion depth is further increased to 255 μm for the 4Ti6TiB_2_ composite (Figure 10h). According to the corrosion depth of the nacre-like composites, it can be concluded that the intergranular corrosion susceptibility of 1Ti9TiB_2_ and 2Ti8TiB_2_ composites is comparable to that of the 2024Al alloys; 3Ti7TiB_2_ and 4Ti6TiB_2_ composites are more resistant to intergranular corrosion than the 2024Al alloys.

## 4. Discussion

### 4.1. Pitting Corrosion

Numerous studies proved that the pitting corrosion reaction of 2024 Al in chloride solutions involves four main stages: the destruction of the passivation film, dissolution of the Al matrix around the intermetallic phases, corrosion gully extension, and dissolution of the intermetallic phases [38,39,40]. Pitting usually tends to occur at the interface between the intermetallic phases and Al matrix. In case of corrosion, the anodic activity is derived from the Al matrix dissolution, which can be described as Equation (1):

(1) $$\mathrm{Al}\to {\mathrm{Al}}^{3+}{+3e}^{-}$$

Meanwhile, the cathode consumes electrons at anodes. In this process, oxygen reduction and hydrogen evolution reactions occur according to Equations (2) and (3), respectively.

(2) $$O_{2}{+H}_{2}{O+4e}^{-}\to {4\mathrm{OH}}^{-}$$

(3) $${2H}_{2}{O+2e}^{-}\to H_{2}{+\mathrm{OH}}^{-}$$

Notably, the formation of free Al^3+^ atoms from Equation (1) is hydrolyzed in chloride solutions, leading to the formation of the corrosion products (Al(OH)_n_^(3−n)^) and a decrease in the pH value (Equation (4)). Furthermore, the Al(OH)_n_^(3−n)^ can be transformed into more stable compounds by Equation (5), resulting in the formation of the passivation oxide film on the corroded surface. However, the destruction of the passivation layer due to the presence of Cl^-^ leads to further corrosion (Equation (6)). Cl^−^ can also promote the dissolution of the Al matrix (Equation (7)).

(4) $${\mathrm{Al}+\mathrm{nH}}_{2}O\to {\mathrm{Al}(\mathrm{OH})}_{n}^{(3-n)}$$

(5) $${2\mathrm{Al}(\mathrm{OH})}_{3}\to {\mathrm{Al}}_{2}O_{3}{+H}_{2}O$$

(6) $${\mathrm{Al}(\mathrm{OH})}_{3}{+\mathrm{nCl}}^{-}\to {\mathrm{Al}(\mathrm{OH})}_{3-n}{\mathrm{Cl}}_{n}{+\mathrm{OH}}^{-}$$

(7) $${\mathrm{Al}}^{3+}{+3\mathrm{Cl}}^{-}\to {\mathrm{AlCl}}_{3}$$

Cl^−^ often plays an important role in the dissolution of the passivation film and formation of pitting. As local electrochemical cells can be formed between the intermetallic phases and Al matrix, galvanic corrosion and hence severe localized pitting in the chloride solutions may appear. Unlike Al alloys, the pitting of nacre-like composites mainly occurred around or within the ceramic-rich layers. In corrosive media, the galvanic cell forms between the ceramic particles and Al matrix because the precipitates at the interface can induce the creation of an island-shaped gully [38,41], as shown in Figure 6(b1–e1). 

A large number of corrosion pits also occurred in the ceramic-rich layers due to the presence of an Al matrix within the ceramic-rich layers (Figure 10(a1–d1)). Compared to the 3Ti7TiB_2_ composite, the pitting within the ceramic-rich layer of the 1Ti9TiB_2_ and 2Ti8TiB_2_ composites is more serious despite its smaller size. Severe pitting corrosion also appeared in the ceramic-rich layer of 4Ti6TiB_2_ composites. So, the corrosion crack depths decrease and then increase as the Ti/TiB_2_ ratio increases due to the presence of pores and Fe-Ti intermetallic phases in the ceramic-rich layer, which significantly increases the susceptibility to pitting and corrosion. As the electrochemical dissolution rate is proportional to the activation energy of the metal ions to dissolve into a solution [41], the relatively loose ceramic-rich layers in the 1Ti9TiB_2_ and 2Ti8TiB_2_ composites allow higher corrosion current density and lower impedance than the 3Ti7TiB_2_ composites. So, the 3Ti7TiB_2_ composite showed the best corrosion resistance. However, a further increase in the Ti/TiB_2_ ratio (4Ti6TiB_2_) can lead to the formation of a large number of Fe-Ti intermetallic phases in the ceramic-rich layers, which accordingly can induce more severe pitting corrosion. 

### 4.2. Intergranular Corrosion

Intergranular corrosion has been reported in Al alloys to occur mainly at grain boundaries and the free zone of the precipitates. As the secondary phase at the grain boundaries is less self-corrosive than the Al matrix, it may act as a cathode in the corrosive medium to induce preferential dissolving of the Al matrix, and thus lead to a severe corrosion of the grain boundaries [29,42,43]. As can be seen in Figure 9, significant intergranular corrosion occurred in the 2024Al alloy. For nacre-like (TiBw-TiB_2_)/2024Al composites, the intergranular corrosion mainly occurred in the Al matrix layers, whereas pitting corrosion dominated in the ceramic-rich layers (Figure 10(a1–d1)). Moreover, the corrosion depth decreases with an increasing ratio of Ti/TiB_2_, which can be attributed to the increased density of the ceramic-rich layers in the composites. The increased density of the ceramic-rich layers was related to the enhanced wettability between ceramic and molten Al with the presence of remaining Ti in the matrix, which increased the densification of the infiltrated composites.

Specially, corrosion propagates along the ceramic-rich layers from the surface of the Al matrix to the interior, as shown in Figure 10e–h. This fact indicates that the ceramic-rich layers provide a corrosion channel for the corrosion medium during intergranular corrosion. Zhao et al. [20] found that the intergranular corrosion susceptibility of TiC/Al composites is closely related to the distribution of the TiC particles. The corrosion resistance of the composites with uniform TiC distribution was superior to those with reticulated or chain-like particle distribution. As a result, the continuity of the TiC particles is the key to the intergranular corrosion susceptibility of the composites. The better the continuity of the TiC particles, the worse the intergranular corrosion resistance of the composites. Similarly, in this work, the nacre-like (TiBw-TiB_2_)/Al composites had a continuous layered ceramic structure, which led to a rapid propagation of intergranular corrosion in the ceramic-rich layer and degraded the corrosion resistance of the composites. 

**Figure 11:**
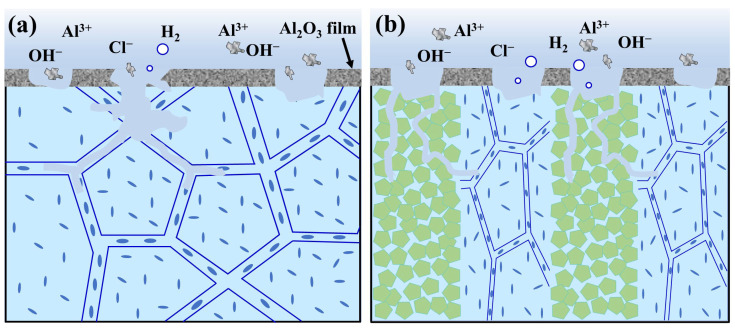
Schematic diagrams of intergranular corrosion mechanisms in the 2024Al alloy and the nacre-like (TiBw-TiB_2_)/Al composites. (**a**) The intergranular corrosion of 2024Al alloys at the grain boundaries; (**b**) nacre-like (TiBw-TiB_2_)/Al composites subjected to intergranular corrosion along the ceramic-rich layers.

Based on the above discussion, the corrosion mechanisms of the nacre-like (TiBw-TiB_2_)/Al composites are schematically illustrated in Figure 11. For the Al matrix layers, when the passivation film on the surface was broken, a high intergranular corrosion susceptibility occurred at the grain boundary precipitates as well as the precipitate-free zones due to the lower corrosion potential. Therefore, the intergranular corrosion occurred at the grain boundaries (Figure 11a). For the ceramic-rich layers, the potential of the coarse Fe-Ti intermetallic phases is more negative than that of the Al matrix, leading to the pitting corrosion. In addition, the continuous ceramic-rich layers provide a propagation path for the intergranular corrosion. Once the precipitates at the grain boundary are preferentially dissolved, intergranular corrosion may rapidly propagate along the ceramic-rich layer (Figure 11b).

## 5. Conclusions

The nacre-inspired (TiBw-TiB_2_)/Al composites were prepared by freeze casting in combination with squeeze casting. The effects of the Ti/TiB_2_ ratio on the microstructure and the corrosion behavior of the composites were systematically studied, and the main conclusions can be drawn as follows: 

(1) With the increase in the Ti/TiB ratio, the content of Fe-Ti intermetallic phases in the composites increased. The Al and ceramic-rich layers showed little change with the Ti/TiB ratio and were about 35 μm and 31.2 μm, respectively. A large amount of a Cu-rich phase remained in the ceramic-rich layer after the solid solution because the ceramic-rich layer inhibited the dissolution of the Cu-rich phase.

(2) The corrosion resistance of nacre-like (TiBw-TiB_2_)/Al composites increased and then decreased with an increasing Ti/TiB_2_ ratio, where the 3Ti7TiB_2_ composite exhibited the best corrosion resistance. Electrochemical tests showed that the 3Ti7TiB_2_ composite had the lowest corrosion current density (15.9 μA/cm^2^), which was 66% lower than that of the 1Ti9TiB_2_ composite.

(3) Unlike the corrosion pits formed in 2024Al alloys, a large number of corrosion products were formed on the surface of the composites, and some cracks were generated around the corrosion products after the electrochemical corrosion test. Although the corrosion products on the surface of the 3Ti7TiB_2_ composite were fewer than those of the 1Ti9TiB_2_ and 4Ti6TiB_2_ composites, they were denser due to the fact that there were relatively fewer cracks around the corrosion products on the surface. In addition, a more stable and thicker passivation film was formed on the surface of the 3Ti7TiB_2_ composite. Therefore, the 3Ti7TiB_2_ composite exhibited the best electrochemical corrosion resistance.

(4) The continuous distribution of ceramic-rich layers was not favorable for the intergranular corrosion resistance of nacre-like (TiBw-TiB_2_)/Al composites, since the ceramic-rich layers can act as corrosion channels. The intergranular corrosion results illustrated that the 3Ti7TiB_2_ composite had the lowest corrosion depth (231 μm), indicating the lowest sensitivity to intergranular corrosion. In addition, the Fe-Ti intermetallic phase and the remaining Cu-rich phase led to pitting corrosion in the ceramic-rich layer, while the Al layer underwent significant intergranular corrosion due to precipitation in the grain boundaries.

## Data Availability

The data related to this work can be obtained from the corresponding author upon reasonable request.
